# 

**Published:** 1912-07-13

**Authors:** 


### OUR BUREAU OF INFORMATION.

### Rules for Correspondents.

1. Every letter, on whatever subject, must be accompanied by the coupon to be cut from the back cover (inside page) of THE HOSPITAL, current issue, and must contain the name and address of the correspondent with pseudonym for publication if desired.

2. Replies by post cannot be given, save under exceptional circumstances, at the Editor's discretion.

3. Letters from Approved Homes in reply to special needs published in the Bureau should state terms and full particulars, and be sent prepaid under cover to the Editor of the Bureau with coupon and name enclosed for identification.

4. Proprietors of homes which have not yet been entered on the List of Approved Homes, but have spare accommodation likely to suit special needs, are invited to write for an application form for registration. The fee for registration is 5s.

5. Special needs of every description relating to those who are sick or in difficulties are made known in these columns free of charge.

6. All communications to be addressed to The Editor of THE HOSPITAL, 28 Southampton Street, Strand, London, W.C., and marked "Bureau of Information."

### Needs and Helpers.

### Pay Hospital for Case of Hernia.

E. B.—A working Englishwoman in Spain is sent to England for a radical cure of hernia. She cannot afford to go into a nursing home or pay surgical fees, but could contribute or give a moderate sum weekly in return for accommodation and treatment. What hospitals in or near London would receive such a case on terms of that kind? The patient's respectability and the genuineness of the circumstances are personally known to the querist, who is a registered medical man.

We have ascertained that the Grosvenor Hospital for Women, Vincent Square, Pimlico, will receive the patient to whom you refer. The terms are 10s. a week in a small ward (containing two to six patients), and this includes the entire expense of nursing and operation. Private wards cost £2 2s. a week. Will you write to the Secretary for an application form?

### Hospital Worker Blind and Deaf.

D. B., Maidstone.—Our visitor reports most warmly and sympathetically. The fact that you lost both sight and hearing through typhoid caught in the performance of your duties in an isolation hospital gives you a strong claim to charitable assistance. We do not advise your trying to join a benefit society and you need not be insured. You should make an effort to procure a pension to supplement your means from one of the blind societies. You must first obtain a certificate of blindness from the Maidstone Hospital. Then write to the secretary of one of the following societies and state your case, and ask them to forward you a form of application to fill in. Perhaps your clergyman or visiting lady or district nurse would help you, as writing must be a great difficulty: Came's Charity, 7 Cannon Street, London, E.C.; Clothworkers' Company, 41 Mincing Lane, London, E.C.; Gardner's Trust for the Blind, 53 Victoria Street, Westminster; Goldsmiths' Company, Foster Lane, E.C. The latter gives pensions to the inhabitants of Kent especially. Let us know how you prosper, and persevere.

### Home Wanted for Convalescent Nurse.

D. E. M. (113A).—We are sure that some fellow nurse will be found to offer you accommodation through the summer for 12s. 6d. or 15s. a week, especially as you are willing to be useful. We will forward replies.

### Home for Incorrigible Girl.

PATIENCE.—On no account let the child you describe go where she will be associated with fallen girls. There is more than one home for children who have shown themselves unamenable to home discipline; but the best we know is St. Helena's Home, Ealing. Write to the Sister Superior. The girls are such as have been guilty of theft or other misdemeanour, and as their faults are frequently due to a faulty environment and bringing up rather than to any radical defect of character, it is found that they respond quickly to good influence and for the most part do well after training. A small weekly payment must be guaranteed.

### Hospital Administration.

### Women on Hospital Boards.

MR. A. E. THOMAS.—We are much interested to know that a lady was elected to the Board of the North-West Hospital (Hampstead General) in 1898, and is still a member of the Board.

### The Cost of Maintaining a Bed.

D. P. OLD GIRLS.—The average cost of maintaining a bed in a London hospital varies from £134 in the case of the London Hospital to between £60 and £100 in smaller hospitals without medical schools. Probably even the smallest figure would be beyond the funds you have to dispose of yearly. Hospitals for children vary in cost per bed between the Victoria Hospital at £67 and the Evelina Hospital at £92. We advise you to choose a good convalescent home where the cost would be far less, or one of the hospitals where children are received for long continued treatment. Write to the Cheyne Hospital, Cheyne Walk, Chelsea, and ask their charge for keeping up a bed through the year. We understand that it is your desire to pay the entire cost.

### Notice to Hospital Secretaries.

We are compiling a List of Institutions at home and abroad receiving paying patients, and shall be glad to have particulars of any accommodation provided for the large class of persons able and willing to contribute towards treatment, though unable to pay its entire cost.

### Letter-Box.

H. P.—We are most grateful to you for your help. The points required in any report of those in need of help are as follows: Age, length of residence in locality, place of birth, occupation of father or husband, previous employment, exact income from all sources, whether member of any religious body, any bodily infirmity, references. All or any of these points may suggest some possibility of working up help, and if this is once understood there will be no reluctance to give information. It is always easier to procure help for persons with some income, however slender to start with.

M. S. (112A).—We have forwarded on some kind offers from Approved Homes and hope to hear soon if one has been accepted. We are compelled to remind our numerous other correspondents of the rule which obliges us to make independent investigation into the management of all homes which we recommend in these columns. The fee for making these inquiries is five shillings, but when once registered no further fee is ever required, and letters are forwarded whenever desired.

### The Queen and King Edward VII.'s Hospital, Cardiff.

After leaving the King Edward VII.'s Hospital, Cardiff, on June 28, her Majesty sent back some flowers, which were distributed amongst the patients. The Matron of the hospital, Miss E. A. Mont Wilson, subsequently wrote to Viscount Stamfordham asking him to be good enough to convey her thanks to the Queen, and received the following gracious reply :—

"Buckingham Palace, July 2, 1912.

"Dear Miss Wilson,—I have shown your letter to the King and Queen, who read it with much satisfaction. I am commanded to thank you for it, and to assure you what a great pleasure it was to their Majesties to visit such a beautiful hospital as King Edward VII.'s at Cardiff.—Yours very faithfully,

"(Signed) Stamfordham.

"Miss E. A. M. Wilson, Matron, King Edward VII.'s "Hospital, Cardiff."

### Appointments.

The Mental Hospitals Committee of the London County Council have appointed Col. R. J. Cooper and Mr. E. G. Easton to be members of the Banstead Sub-Committee, and Mr. E. G. Easton to be a member of the Epileptic Colony and Horton Estate Sub-Committee.

The following appointments have been made at Guy's Hospital: Mr. C. J. Symonds, M.D., M.S., F.R.C.S., on his retirement from the post of senior surgeon, to be consulting surgeon to the hospital; Mr. F. J. Steward, M.D., F.R.C.S., senior assistant surgeon, to the full staff; and Mr. Robert Davies-Colley, M.A., M.C. (Cantab.), F.R.C.S., as fifth assistant surgeon.

Dr. T. Strain, M.D., medical officer, Barnes, has been appointed medical officer of health and medical officer of the schools, Hounslow. Dr. Strain, who is a graduate of Glasgow, and holds the Cambridge Diploma in Public Health, has been medical superintendent of the Isolation Hospital and of the Tuberculosis Dispensary, Barnes. His past hospital appointments include those of house physician at the Kensington General Hospital and clinical assistant at the Royal Hospital for Sick Children, Glasgow. Dr. Strain gained the appointment, for which there were fifty-five candidates, by a casting vote.

### Gifts and Bequests.

The Chelsea Hospital for Women has received from an anonymous donor, "In Memoriam," the sum of £500 for its Rebuilding Fund.

Mrs. Louisa Darell-Brown has left £500 each to the East Sussex County Hospital, Hastings, and to the Buchanan Homœopathic Hospital, St. Leonards.

Mr. Arthur Bolden Davison, formerly Registrar of the Chancery Division in Ireland, has left £1,000 each to the Brompton Cancer Hospital, the Brompton Hospital for Consumption, the National Hospital for the Paralysed and Epileptic, and the Hospital for Diseases of the Skin.

One Thousand Guineas have been received from the Rev. Canon Wilson, of Carlisle, trustee of the late Mrs. Morrison, of St. Helen's, Hastings, to name a bed in the Eversfield Hospital for Consumption and Diseases of the Chest at St. Leonards-on-Sea, in memory of her son, to be named the "George Edward Morrison Bed." Also £25 has been received from the Worshipful Company of Drapers towards the Extension Fund.

### A DOCTOR'S BEQUEST.

Dr. Herbert Nankivell, of Bournemouth, consulting physician to the Hahnemann Convalescent Home, and author of several medical works, who died on April 6, aged sixty-eight, left estate of the gross value of £28,750, with net personalty £13,990. He bequeathed £400 to the Hahnemann Convalescent Home for fully endowing a bed partially endowed in memory of his former wife Etheldreda.

### Buildings Contemplated.

Arbroath.—The Infirmary Committee have under consideration plans prepared by Mr. Hugh Gavin for new buildings estimated to cost £14,000.

Argentina.—The *Boletin Oficial* publishes a decree authorising the Superintendent of Military Construction to erect an hospital in Campo de Mayo, Argentina, at a cost of about £39,370.

Benfieldside.—The Governors of the Murray Commemoration and Memorial Hospital have accepted a tender of £9,296 for new hospital premises. The highest of twenty-one tenders amounted to £11,538.

Blackhill.—Mr. J. Eltringham is architect for the proposed Richard Murray Hospital to be built at a tendered cost of £12,400.

Bolton.—The Committee invite tenders for the erection of an Edward VII. Memorial Nurses' Home at the infirmary. Particulars may be obtained from the architects, Messrs. Henderson and Brown, of Bolton, on deposit of £2 2s.

Canterbury.—It is proposed to erect at the Canterbury Workhouse a laundry, boiler-house, and chimney shaft. The architect is Mr. F. H. Doré, of Canterbury. Tenders are now under consideration.

Chester.—For the extension and renovation of Chester Infirmary an effort is being made to raise £40,000.

Dumbartonshire.—The County Council have decided to purchase 119 acres near Drumchapel at a cost of £3,250 as a site for a smallpox hospital.

Dundee.—Tenders are being considered for 1,550 superficial yards of composite jointless flooring to be laid at King's Cross Hospital for Dundee Town Council.

Edmonton.—The Joint Hospital Board have decided to erect a residence for the medical superintendent at an estimated cost of £1,235.

